# The Role of Cadence and Torque in Fatigue-Related Power Output Decline in Cycling’s Grand Monuments

**DOI:** 10.3390/sports13110406

**Published:** 2025-11-12

**Authors:** Alejandro Javaloyes, Jose Luis Sánchez-Jiménez, Iván Peña-González, Manuel Moya-Ramón, Manuel Mateo-March

**Affiliations:** 1 Department of Sport Sciences, Sports Research Centre, Miguel Hernández University, 03202 Elche, Spain; ajavaloyes@umh.es (A.J.); ipena@umh.es (I.P.-G.); mmoya@umh.es (M.M.-R.); manuel.mateom@umh.es (M.M.-M.); 2 Research Group in Sports Biomechanics (GIBD), Department of Physical Education and Sports, University of Valencia, 46010 Valencia, Spain

**Keywords:** cycling performance, cadence, torque, fatigue, durability, Monuments

## Abstract

This study examined the effects of cadence and torque on fatigue-related power output (PO) decline in professional cyclists during the Five Monuments, comparing top-5 finishers with cyclists ranked from 6th to 30th. Retrospective data from 64 male cyclists (top-5 *n* = 14, top-30 *n* = 42) in the 2021–2023 Five Monuments were analyzed. PO, cadence, and torque profiles were constructed for 10 s, 1 min, 5 min, and 20 min maximal mean power efforts and after 30–60 kJ·kg^−1^ workloads. Repeated-measures ANOVA assessed group differences, and Pearson correlations evaluated variable relationships under fatigue. Top-5 finishers exhibited higher PO (e.g., 20 min: *p* = 0.003; 60 kJ·kg^−1^: *p* < 0.001) and torque (e.g., 20 min at 60 kJ·kg^−1^: *p* < 0.001) compared to cyclists ranked 6th to 30th. They also displayed lower cadence during 10 s efforts at 50–60 kJ·kg^−1^ (*p* = 0.008). Top-5 cyclists maintained stable PO and torque beyond 60 kJ·kg^−1^, whereas the top-30 group showed significant declines (*p* < 0.001). Torque was strongly correlated with PO (r = 0.6–0.9, *p* < 0.001), while cadence showed a weaker correlation (r = 0.1–0.5). Top-5 cyclists show greater durability, sustaining higher torque and power output during prolonged efforts with minimal cadence changes. These biomechanical traits distinguish elite performers in the Five Monuments and underscore the value of training for torque sustainability and fatigue resistance.

## 1. Introduction

Professional cycling demands a combination of physiological, biomechanical, and tactical excellence, particularly in the most prestigious one-day races, the Five Monuments of Cycling—Milan-San Remo, Tour of Flanders, Paris-Roubaix, Liège-Bastogne-Liège, and Il Lombardia. These races are characterized by extreme distances (ranging from approximately 240 to 300 km), intense workloads, and challenging terrain—often hilly or featuring cobblestone sections—making them a benchmark for evaluating the capabilities of elite cyclists [1]. Success in these events requires not only peak power output (PO) but also the ability to sustain performance under increasing fatigue [2]. This ability to maintain high PO despite accumulating fatigue, known as durability, is a key determinant of performance in professional cycling [3]. Previous studies have shown that World Tour cyclists outperform their ProTeam counterparts in terms of mean maximal power (MMP) and durability, especially under workloads exceeding 7.5 kJ·kg^−1^ [4]. This superior durability is attributed to training adaptations, such as improved metabolic efficiency (e.g., shifting to a more efficient and preferential substrate oxidation), and optimized recovery strategies, which enable these athletes to sustain higher outputs over prolonged efforts [5,6]. Although much research has focused on multi-stage events like the Grand Tours [7,8,9,10,11], studies examining the specific demands of these Monuments are limited. These races present unique physiological and biomechanical challenges, as they condense extreme workloads into a single, prolonged day of competition—typically lasting six hours or more—with repeated high-intensity efforts and technical demands such as cobblestone sections. Unlike Grand Tours, where riders must manage cumulative fatigue over consecutive days, the Monuments require cyclists to optimize performance in one decisive event [12].

While PO is well-studied, less is known about how cadence—the pedaling rate measured in revolutions per minute—and torque—the rotational force applied to the crank—interact with fatigue and influence success in these events [3,13]. Cadence and torque distribution play a crucial role in cycling efficiency, as they determine the neuromuscular and metabolic demands placed on the rider during prolonged efforts [14,15]. Biomechanical models emphasize the role of muscles as elastic energy return systems, which not only produce torque but also store and release energy efficiently, reducing the torque required for pedaling, enhancing movement economy, and delaying fatigue accumulation [16]. In cycling, this suggests that optimizing torque throughout the pedaling cycle can help maintain power output while reducing fatigue-related declines in cadence and neuromuscular efficiency. This model complements previous research indicating that an optimal balance between cadence and torque can minimize energy expenditure while sustaining high PO during repeated high-intensity efforts [17]. In prolonged endurance events, fatigue-induced changes in cadence and torque application can lead to alterations in muscle recruitment patterns, potentially affecting performance outcomes [18]. For instance, reductions in cadence and increases in torque have been observed as fatigue progresses, potentially increasing muscular strain and energy cost [19]. Conversely, an excessive reliance on high cadences may lead to greater cardiovascular stress, further challenging the ability to sustain performance [20]. These biomechanical adjustments under fatigue may be particularly relevant in the Monuments, where terrain variability, cobbled sections, and steep climbs require continuous adaptations in pedaling mechanics [2]. Despite the recognized importance of cadence and torque in cycling performance, their role in one-day races remains underexplored. Understanding how professional cyclists regulate these parameters in response to increasing workload and fatigue could provide new insights into the determinants of success in professional cycling. Therefore, the aim of this study is to determine how torque and cadence change when fatigue produces a power output decline. We hypothesize that cadence will decrease in association with reductions in power output under fatigued conditions.

## 2. Materials and Methods

### 2.1. Participants

This study included 64 professional male cyclists, classified by performance level into two groups, based on a previous study [21]: those who achieved a top-5 finish (*n* = 14) and those ranked between 6th and 30th positions (top-30; *n* = 42). Table 1 details the characteristics of the groups. Data were collected from single-day cycling races, referred to as the Monuments (Milan-San Remo, Tour of Flanders, Paris-Roubaix, Liège-Bastogne-Liège, and Il Lombardia), conducted between 2021 and 2023. For comparison purposes, we specifically included Monument races where cyclists secured a top-30 placement, as these performances are likely to reflect maximal effort aimed at achieving victory, in contrast to lower-ranked finishes where team strategies or individual priorities might lead to submaximal efforts. This study adhered to the principles outlined in the Declaration of Helsinki, with all participants providing written informed consent. The protocol was approved by the Institutional Review Board.

### 2.2. Sample Estimation and Justification

The required sample size to detect differences in PO, cadence, and torque between groups was estimated using a repeated-measures ANOVA design (within–between interaction), considering two groups and six repeated measurements. Based on an expected medium effect size (Cohen’s f = 0.3) considering the results of a previous study [20], a statistical power of 95%, and a significance level of 5%, the estimated minimum sample size was 14 participants. The calculation was performed using G*Power 3.1 software (University of Düsseldorf, Düsseldorf, Germany).

### 2.3. Study Design

The present study followed an observational, retrospective design. Race files were collected retrospectively along with race results and descriptive characteristics (e.g., age, body mass, and height). Each race file was then analyzed to extract power output, torque, and cadence data, which were used to construct performance profiles from fresh to fatigued states.

### 2.4. Dataset

Athletes provided competition data recorded through their performance tracking devices (e.g., Garmin, Lenexa, KA, USA; Wahoo Fitness, Atlanta, GA, USA), which were exported in “.fit” format. The tracking devices were employed only for data recording purposes. The dataset included variables such as time, location, distance, elevation, PO, cadence and calculated torque sampled and processed at 1 s intervals (1 Hz). No significant effect of the cycling computer was expected under steady-load conditions or when analyzing mean maximal values [22].

### 2.5. Measures

PO data were reviewed visually to identify and address potential anomalies, commonly referred to as “spikes”. Each PO data point was scrutinized for irregular or extreme values using dedicated software (WKO5 Build 576; TrainingPeaks LLC, Boulder, CO, USA). Any identified anomalies were manually corrected utilizing the software’s Data Spike ID and FIX chart, particularly for abrupt, non-linear changes in the maximum PO values recorded for each effort duration. The analysis included only PO data captured during the races, explicitly excluding warm-ups, cool-downs, and recovery activities. All cyclists used their team-provided bicycles, which were individually configured to their preferences.

Performance was evaluated by constructing power profiles across all races. The MMP values for 10 s, 1 min, 5 min, and 20 min were calculated for each cyclist using all available race data. These effort durations are commonly employed in cycling performance analysis, as reported in a recent systematic review [23], due to their distinct physiological emphasis. Once these best efforts were identified, the corresponding mean cadence and torque values were extracted to create cadence and torque profiles derived from the record power profile. These additional profiles were used to identify whether potential reductions in power output with increasing fatigue were primarily associated with changes in torque or cadence. Torque was calculated using the equation: Torque = PO ÷ (cadence × π ÷ 30), as described by Gardner et al. [3], and cadence was directly obtained from files. In addition, MMP, cadence, and torque values were calculated for the aforementioned durations after varying levels of accumulated work (30, 40, 50, and 60 kJ·kg^−1^). The cyclist’s maximal performance before the accumulation of substantial fatigue was defined as the “fresh” state. A compound score (W^2^·kg^−1^) was calculated as the product of absolute power output for the 5 min mean maximal value (W) and relative power output (W·kg^−1^), providing an integrated indicator of cycling performance [24].

### 2.6. Statistical Analysis

All analyses were performed using R software (R Core Team, Vienna, Austria) through RStudio (version 4.2.0). Data are reported as mean ± standard deviation (SD). Normality of the data was assessed using the Shapiro–Wilk test. Homogeneity of variances was assessed using Levene’s test, and Welch’s *t*-test was applied if variances were unequal. Separate repeated-measures ANOVAs with Bonferroni post hoc adjustments were conducted for each effort duration (i.e., separate analyses for 10 s, 1 min, 10 min, and 20 min). For each duration, MMP in the fresh state was compared to MMP fatigued states after 30, 40, 50, and 60 kJ·kg^−1^ of energy expenditure. This approach allowed independent evaluation of changes in power, cadence, and torque across fatigue levels for each specific duration, rather than combining all durations into a single model. Pearson correlations were computed to explore relationships between power, cadence, and torque across different workloads and to assess the consistency of these variables under fatigue. The strength of correlations was interpreted based on conventional thresholds: weak (r = 0.10–0.29), moderate (r = 0.30–0.49), and strong (r ≥ 0.50). Statistical significance was set at α = 0.05.

## 3. Results

**Figure 1:**
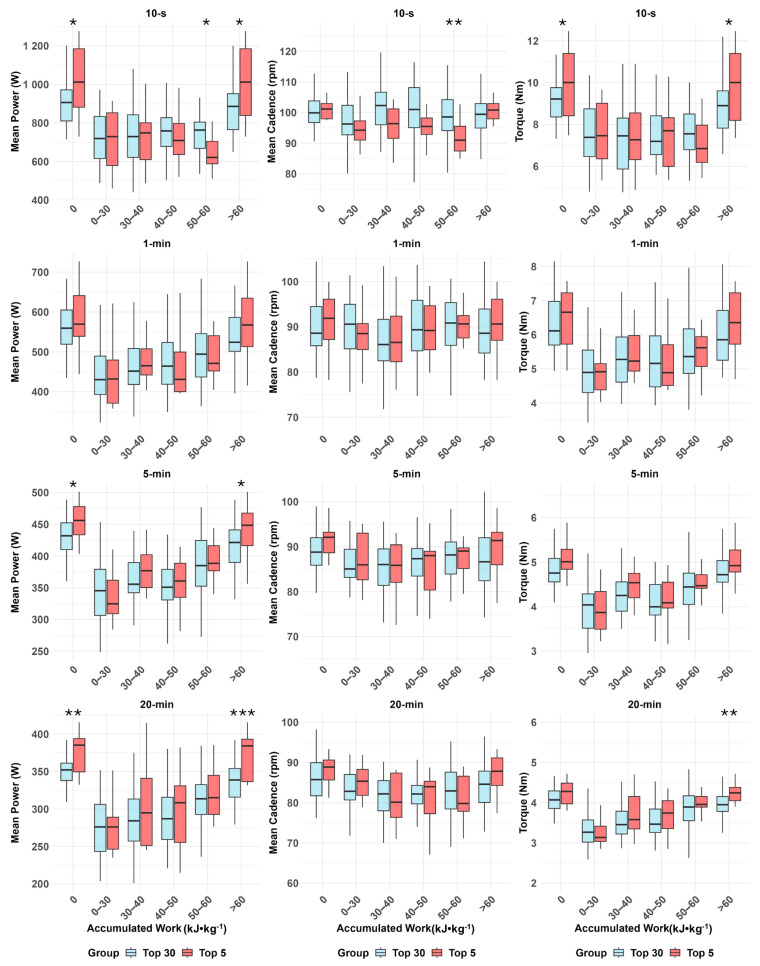
Mean maximal power (MMP), cadence (rpm), and torque (Nm) during the record power profile durations (10 s, 1 min, 5 min, and 20 min) in Monument races, comparing top-5 and top-30 cyclists in the fresh (0 kJ·kg^−1^) state and after accumulated energy expenditures of 30, 40, 50, and 60 kJ·kg^−1^. Differences between top-5 and top-30 cyclists: * *p* < 0.05, ** *p* < 0.01, *** *p* < 0.001.

Figure 1 and Table 2 show the MMP with corresponding cadence and torque profiles across the “fresh” and after varying levels of energy expenditure of Five Monument races compared between top-5 and top-30 cyclists.

### 3.1. Power Profile

Intergroup analysis revealed that top-5 cyclists achieved significantly higher MMP than top-30 cyclists for 10 s (*p* = 0.030), 5 min (*p* = 0.030), and 20 min efforts (*p* = 0.003), as well as after 60 kJ·kg^−1^ for 10 s (*p* = 0.014), 5 min (*p* = 0.020), and 20 min efforts (*p* < 0.001). No significant intergroup differences were observed for 1 min efforts at any accumulated work levels (*p* > 0.05).

In intragroup analysis, the top-5 group showed significant PO reductions from 0 to 60 kJ·kg^−1^ across all effort durations (*p* < 0.001) but maintained stable PO beyond this threshold (*p* > 0.05). Conversely, the top-30 group exhibited significant PO reductions compared to their MMP at all accumulated work levels for all effort durations (*p* < 0.001).

### 3.2. Cadence Profile

Intergroup analysis revealed that during 10 s efforts, top-5 cyclists exhibited lower cadence than top-30 cyclists at 50–60 kJ·kg^−1^ (*p* = 0.008), with no differences at other levels (*p* > 0.05). No significant differences in cadence between top-5 and top-30 cyclists for 1 min, 5 min, or 20 min efforts at any accumulated work level (*p* > 0.05).

Intragroup analysis showed a cadence reduction in 10 s efforts for top-5 at 50–60 kJ·kg^−1^ (*p* = 0.002), while top-30 cyclists exhibited no significant changes at any accumulated work level (*p* > 0.05). No significant intragroup changes were observed for 1 min efforts (*p* > 0.05). In 5 min efforts, cadence decreased at 30–40 kJ·kg^−1^ (top-5: *p* = 0.020; top-30: *p* = 0.001), while in 20 min efforts, reductions occurred from 30 to 60 kJ·kg^−1^ (top-5: *p* = 0.003; top-30: *p* < 0.001).

### 3.3. Torque Profile

Intergroup analysis showed that top-5 cyclists produced significantly higher torque than top-30 cyclists for 10 s at MMP efforts (*p* = 0.026) and after 60 kJ·kg^−1^ (*p* = 0.016), as well as in 20 min efforts after 60 kJ·kg^−1^ (*p* < 0.001). No intergroup differences were observed for 1 min or 5 min efforts at any accumulated work level (*p* > 0.05).

In the intragroup analysis, both groups showed significant torque reductions in 10 s efforts at all accumulated work levels up to 60 kJ·kg^−1^ (*p* < 0.001) but remained stable beyond this point (*p* > 0.05). For 5 min efforts, significant reductions were observed at 0–30, 30–40, 40–50, and 50–60 kJ·kg^−1^ (*p* < 0.001), with no changes beyond 60 kJ·kg^−1^ (*p* > 0.05). Top-5 cyclists showed significant torque reductions during 20 min efforts at 0–30 (*p* < 0.001), 30–40 (*p* < 0.001), 40–50 (*p* < 0.001), and 50–60 kJ·kg^−1^ (*p* = 0.018), but remained stable beyond 60 kJ·kg^−1^ (*p* > 0.05). In contrast, top-30 cyclists exhibited torque reductions at all accumulated work levels, including beyond 60 kJ·kg^−1^ (*p* = 0.005).

### 3.4. Correlations

Moderate and strong correlations (r = 0.5–0.9, *p* < 0.001, Table 3) were found between PO and torque, whereas correlations between PO and cadence were weaker (r = 0.1–0.5, *p* < 0.05). Significant correlations (*p* < 0.05) were found between both MMP and fatigued PO and both MMP and fatigued torque. In contrast, correlations between power and both MMP and fatigued cadence in the 1 min effort were not significant (r = 0.1, *p* > 0.05). Similarly, in the 5 min effort, the correlation between fatigued PO and MMP cadence was not significant (r = 0.2, *p* = 0.106). Finally, the strongest correlations with torque were observed for the shortest effort durations (10 s and 1 min), with r values generally decreasing as effort duration increased.

## 4. Discussion

Durability, defined as the ability to maintain or even enhance performance under fatigue, is a key determinant of elite cycling performance [2,3]. Understanding how cyclists sustain torque and PO under accumulated fatigue is critical for explaining success in the most demanding one-day races, the Monuments. Therefore, the aim of this study was to determine how torque and cadence change when fatigue, quantified as accumulated work, produces a decline in power output. This study provides new insights into how the best cyclists, classified as the top-5, outperform their peers by demonstrating superior PO and torque maintenance, accompanied by distinct cadence adjustments under conditions of accumulated fatigue. The results reveal that the top-5 consistently reach their MMP during the most critical phases of the race, typically after accumulating 60 kJ·kg^−1^ of work.

The present findings show that top-5 cyclists achieved significantly higher MMP and torque values than top-30 cyclists during short (10 s) and long (5 min and 20 min) efforts, particularly after accumulating 60 kJ·kg^−1^ of work. Beyond this threshold, the top-5 group maintained stable PO and torque, whereas the top-30 group exhibited a progressive decline across all workloads. This pattern highlights a superior ability among the best cyclists to preserve mechanical performance under fatigue, a key feature of durability. The lack of significant intergroup differences in 1 min efforts suggests that this duration involves a mixed contribution of anaerobic and aerobic energy systems, resulting in comparable performance between groups. In contrast, 5 and 20 min efforts depend primarily on sustained aerobic power and the capacity to maintain torque under high metabolic stress, favoring athletes with greater fatigue resistance. Cadence behavior further supports these observations. While the top-5 cyclists generally maintained a stable cadence, specific reductions were observed during high-intensity efforts—particularly 20 min efforts beyond 30 kJ·kg^−1^ and 10 s efforts between 50 and 60 kJ·kg^−1^. These adjustments likely represent a conscious or automatic strategy to optimize torque and mechanical efficiency when fatigue compromises muscle contractile capacity. Together, these results indicate that elite cyclists rely on nuanced biomechanical regulation to sustain effective power production in fatigued conditions.

The superior ability of top-5 cyclists to sustain higher torque and power output (PO) under fatigue can be explained by a combination of physiological, biomechanical, and tactical factors [4,25]. From a physiological perspective, advanced metabolic efficiency enables these athletes to utilize energy substrates more effectively during high-intensity workloads [6]. Enhanced muscle buffering capacity further delays fatigue by mitigating the accumulation of hydrogen ions (H^+^), which otherwise impair muscle contractile function [26]. These adaptations are essential for maintaining performance during the decisive phases of Monument races, where repeated high-intensity efforts are required. The predominance of between-group differences in longer efforts (5- and 20 min) and, to a lesser extent, in short sprints (10 s), but not in intermediate efforts (1 min), is consistent with previous findings on the distinct physiological determinants of endurance versus mixed-energy efforts [7,25]. Sustained aerobic power and the ability to maintain torque under elevated metabolic stress characterize well-trained endurance athletes, explaining the advantage of the top-5 group in longer durations. Conversely, 1 min efforts rely on both anaerobic and aerobic metabolism, generating a balanced physiological demand that may mask differences between performance levels. This transitional nature of 1 min efforts likely accounts for the absence of intergroup disparities observed in the present study. The cadence modulation patterns identified here provide new insights into biomechanical regulation during fatigue, complementing prior literature that has rarely addressed this aspect. While the top-5 cyclists maintained relatively stable cadence across most conditions, specific reductions emerged during 20 min efforts beyond 30 kJ·kg^−1^ and in 10 s efforts between 50 and 60 kJ·kg^−1^. These targeted adjustments likely reflect a biomechanical strategy aimed at optimizing torque production and mechanical efficiency as fatigue accumulates. Although not all effort durations displayed significant cadence differences, these findings suggest distinct neuromuscular adaptations that may contribute to optimized force application under fatigue [27]. Furthermore, the stabilization of torque beyond 60 kJ·kg^−1^ observed in top-5 cyclists contrasts with the continuous torque decline seen in the top-30 group. This capacity to preserve torque under fatigue supports earlier evidence emphasizing the importance of mechanical efficiency and muscular resilience in elite endurance performance [2,4,25]. Collectively, these findings extend existing knowledge by linking durability and biomechanical optimization, highlighting their combined role in enabling sustained high-level performance in professional cycling.

This study has several limitations. First, its observational design precludes causal inferences, and the aggregation of data from the Five Monuments—each with distinct physiological and tactical demands—may limit generalizability. Second, the absence of data on nutrition or hydration status represents a potential confounding factor that could influence performance outcomes. Finally, the reliance on calculated rather than directly measured torque (Torque = PO ÷ (cadence × π ÷ 30)) may introduce estimation errors.

Future research should aim to validate these findings with interventional designs that can better isolate causal mechanisms underlying fatigue resistance and torque preservation. Incorporating direct measures of muscle activation, metabolic responses, and biomechanical efficiency would allow a more precise characterization of the physiological adaptations distinguishing top-performing cyclists. Expanding the dataset to include women’s professional races and a broader range of competitive levels would also strengthen the ecological validity of the conclusions.

## 5. Conclusions

The results of this study suggest that the top-5 cyclists in Monument races sustain higher torque and PO compared to those ranked from 6th to 30th, particularly during longer efforts (5 and 20 min) and after accumulating 60 kJ·kg^−1^ of work. These cyclists also show minimal reductions in cadence, emphasizing their superior biomechanical efficiency under fatigue. The ability to maintain torque while minimizing cadence adjustments likely contributes to their enhanced durability, enabling better performance in critical race phases. These findings highlight the key role of torque and cadence modulation in determining success in one-day cycling events, providing actionable insights for training and performance optimization.

## Figures and Tables

**Table 1:** Descriptive characteristics of the cyclists included in the study based on the final race classification (top-5 vs. top-30 cyclists).

Characteristic	Top-5 (*n* = 14)	Top-30 (*n* = 42)	95% CI Bottom	95% CI Top	*p* Value (*d*)
Body mass (kg)	70.6 ± 6.8	70.9 ± 5.6	−4	3.3	0.864 (0.05)
Height (cm)	1.82 ± 0.05	1.81 ± 0.05	−0.020	0.050	0.382 (0.2)
BMI (kg·m^−2^)	21.2 ± 1.1	21.7 1.4	−1.3	0.4	0.276 (0.3)
Result in the race	3 ± 2	18 ± 7	−19	−11	**<0.001 (2.3)**
Average power (W)	259 ± 39	254 ± 28	−14	24	0.614 (0.2)
Peak Power (W)	1198 ± 195	1185 ± 142	−84	109	0.795 (0.1)
Normalized Power (W)	316 ± 33	308 ± 25	−9	25	0.357 (0.3)
Compound Score (W^2^·kg^−1^)	3443 ± 523	3063 ± 518	59	701	**0.021 (0.7)**
Work (kJ)	5902 ± 765	5587 ± 552	−148	640	0.353 (0.5)

The comparisons between groups were conducted using Student’s *t*-test for parametric variables and the Mann–Whitney U test for non-parametric variables. Data are presented as mean ± SD. *d* = effect size; 95% CI = 95% confidence interval.

**Table 2:** Descriptive statistics (Mean ± SD) for power output, torque, and cadence by group (Top 5 vs. Top 30) across effort duration and fatigue levels.

Effort	Accumulated Work (kJ·kg^−1^)	Top 5			Top 30		
		Power Output (W)	Torque (N·m)	Cadence (rpm)	Power Output (W)	Torque (N·m^−1^)	Cadence (rpm)
10 s	0	1016 ± 180	10 ± 2	101 ± 3	919 ± 125	9 ± 1	101 ± 7
	0–30	720 ± 154	8 ± 2	95 ± 6	729 ± 134	8 ± 1	97 ± 9
	30–40	726 ± 146	8 ± 2	97 ± 10	736 ± 147	7 ± 2	102 ± 10
	40–50	721 ± 142	8 ± 2	96 ± 8	752 ± 115	7 ± 1	101 ± 9
	50–60	657 ± 112	7 ± 1	93 ± 7	741 ± 99	8 ± 1	99 ± 8
	>60	1004 ± 192	10 ± 2	101 ± 3	883 ± 143	9 ± 1	99 ± 8
1 min	0	589 ± 83	6 ± 1	91 ± 6	565 ± 58	6 ± 1	90 ± 7
	0–30	437 ± 78	5 ± 1	88 ± 6	443 ± 74	5 ± 1	90 ± 7
	30–40	471 ± 51	5 ± 1	87 ± 8	462 ± 70	5 ± 1	87 ± 8
	40–50	458 ± 73	5 ± 1	89 ± 6	477 ± 80	5 ± 1	90 ± 8
	50–60	488 ± 57	5 ± 1	89 ± 7	500 ± 81	6 ± 1	90 ± 7
	>60	577 ± 90	6 ± 1	91 ± 7	533 ± 66	6 ± 1	89 ± 7
5 min	0	450 ± 38	5 ± 0	90 ± 6	429 ± 29	5 ± 0	89 ± 6
	0–30	341 ± 47	4 ± 1	87 ± 6	341 ± 48	4 ± 1	86 ± 6
	30–40	378 ± 31	4 ± 0	85 ± 7	362 ± 35	4 ± 0	85 ± 5
	40–50	352 ± 48	4 ± 1	85 ± 6	353 ± 39	4 ± 0	87 ± 5
	50–60	396 ± 39	5 ± 0	87 ± 4	384 ± 51	4 ± 1	88 ± 5
	>60	444 ± 40	5 ± 0	89 ± 6	417 ± 36	5 ± 0	87 ± 6
20 min	0	374 ± 30	4 ± 0	88 ± 4	351 ± 22	4 ± 0	86 ± 5
	0–30	281 ± 48	3 ± 0	85 ± 4	276 ± 43	3 ± 0	83 ± 6
	30–40	303 ± 55	4 ± 1	81 ± 6	287 ± 42	4 ± 0	81 ± 6
	40–50	298 ± 49	4 ± 1	81 ± 7	291 ± 41	4 ± 0	82 ± 5
	50–60	322 ± 33	4 ± 0	81 ± 6	314 ± 34	4 ± 0	83 ± 6
	>60	369 ± 30	4 ± 0	87 ± 5	336 ± 26	4 ± 0	85 ± 6

**Table 3:** Correlations between mean maximal power (MMP), cadence, and torque during fresh and fatigued states in top-5 and top-30 cyclists in Monument races.

Condition	Variable Compared (Moment)	10 s r (*p* Value)	1 min r (*p* Value)	5 min r (*p* Value)	20 min r (*p* Value)
Power (MMP)	Cadence (MMP)	**0.3 (0.028)**	0.1 (0.464)	**0.3 (0.032)**	**0.4 (<0.001)**
	Cadence (Fatigued)	**0.3 (0.015)**	0.1 (0.555)	**0.3 (0.020)**	**0.4 (0.002)**
	Torque (MMP)	**0.9 (<0.001)**	**0.8 (<0.001)**	**0.7 (<0.001)**	**0.6 (<0.001)**
	Torque (Fatigued)	**0.9 (<0.001)**	**0.7 (<0.001)**	**0.6 (<0.001)**	**0.6 (<0.001)**
Power (Fatigued)	Cadence (MMP)	**0.3 (0.025)**	0.1 (0.517)	0.2 (0.106)	**0.3 (0.010)**
	Cadence (Fatigued)	**0.4 (0.001)**	0.1 (0.591)	**0.4 (0.007)**	**0.5 (<0.001)**
	Torque (MMP)	**0.8 (<0.001)**	**0.6 (<0.001)**	**0.6 (<0.001)**	**0.5 (<0.001)**
	Torque (Fatigued)	**0.9 (<0.001)**	**0.9 (<0.001)**	**0.7 (<0.001)**	**0.7 (<0.001)**

Fatigued = After 60 kJ·kg^−1^. MMP = Mean Maximal Power.

## Data Availability

The data presented in this study are available on request from the corresponding author due to privacy restrictions.
